# Inhibition of the T2R/α-Defensin Pathway Mediates *Nauclea officinalis*-Induced Intestinal Barrier Dysfunction and Microbiota Alterations

**DOI:** 10.3390/toxics14010099

**Published:** 2026-01-21

**Authors:** Xiaoman Li, Yao Yi, Tegele Si, Lianqian Wang, Zhiyong Hu, Jiayue Xiong, Xuemei Bao, Jun Jun, Sachurula Bao, Xiaoping Ji, Minghai Fu

**Affiliations:** 1 Engineering Research Center of Tropical Medicine Innovation and Transformation of Ministry of Education, Hainan Provincial Key Laboratory for Research and Development of Tropical Herbs, School of Pharmacy, Hainan Medical University, Haikou 571199, China; 2 Key Laboratory for Quality Control of Traditional Chinese Medicine (Mongolian Medicine), School of Mongolian Medicine, Inner Mongolia Minzu University, Tongliao 028000, China; 3 School of Traditional Chinese Medicine, Liaoning University of Traditional Chinese Medicine, Shenyang 110847, China

**Keywords:** *Nauclea officinalis*, T2R, α-defensin, gut microbiota, intestinal barrier

## Abstract

Clinical reports have shown that administration of *Nauclea officinalis* (Danmu in Chinese, DM) preparations may cause significant gastrointestinal discomfort. This study aimed to systematically evaluate the adverse effects of DM and its primary active constituent, strictosamide, on gastrointestinal motility, intestinal barrier integrity, and gut microbiota homeostasis. Furthermore, we sought to investigate the potential role of the bitter taste receptor (T2R) signaling pathway in mediating these effects. In vitro cell cultures and ex vivo intestinal tissues were employed to assess cell viability and molecular alterations. In vivo studies involved short-term (2 weeks) gavage of DM (0.54 and 1.08 g/kg) and long-term (16 weeks) intervention (0.4, 0.8, and 1.2 g/kg) in rodents. Evaluations included histopathological examination, serum levels of cytokines and oxidative stress markers (ELISA), expression of tight junction proteins (Western blot and qPCR), and 16S rDNA sequencing of cecal microbiota. Mechanistic analyses focused on α-defensin secretion and T2R-associated gene and protein expression. Administration of DM resulted in significant gastrointestinal dysfunction, characterized by delayed intestinal propulsion and increased gastric retention. Dose-dependent histopathological damage, disruption of the intestinal barrier (reduced occludin and claudin-1 expression), and elevated levels of pro-inflammatory cytokines (IL-6, TNF-α, and IL-1β), oxidative stress markers (MDA, SOD, and GSH-Px), and immune mediators (IFN-γ) were observed. Gut microbiota analysis revealed dysbiosis, marked by a decline in beneficial genera (e.g., Mucispirillum, Butyricicoccus, Roseburia) and an increase in potentially pathogenic bacteria (e.g., Citrobacter, Helicobacter). Mechanistically, DM suppressed α-defensin secretion and downregulated the expression of TAS2R108, TAS2R138, and Gα-gustducin both in vitro and in vivo. DM and strictosamide disrupt gut microbiota composition and compromise intestinal barrier function, likely through inhibition of the T2R/α-defensin pathway. These findings provide important mechanistic insights into drug-induced gastrointestinal toxicity and underscore the potential risks associated with prolonged use of DM-containing preparations.

## 1. Introduction

*Nauclea officinalis* (commonly known as Danmu in China, DM), derived from the dried stems and roots of *Nauclea officinalis* Pierre. ex Pitard, is characterized by its intense bitterness and cold nature [1]. Modern pharmacological studies have identified abundant monoterpene indole alkaloids such as strictosamide and 3-epi-dihydrocorymine, as well as triterpenoids, exhibiting broad-spectrum antibacterial, anti-influenza virus, and immunomodulatory activities. Clinically, traditional DM preparations—including syrups, tablets, and capsules—are widely used for respiratory infections and skin inflammation [2]; however, adverse gastrointestinal reactions, such as urticaria (0.23%), abdominal discomfort (0.38%), vomiting (1.08%), and diarrhea (1.29%), have been reported, with unclear mechanisms [3].

The intestinal barrier, crucial for maintaining homeostasis, is the first line of defense against luminal insults. Its integrity relies on physical (e.g., epithelial tight junctions), chemical, immune, and biological (gut microbiota) components [4]. Disruption of this barrier, often triggered by inflammatory cytokines or microbial dysbiosis, increases permeability and can lead to gastrointestinal inflammation [5,6,7,8,9]. Interestingly, bitter taste receptors (T2Rs) expressed in gut epithelial cells have emerged as key sensors mediating diet–microbiota–host crosstalk [10,11,12]. Ligand binding activates Gα-gustducin, stimulating Paneth cells to secrete α-defensins and maintain a *Firmicutes* and *Bacteroidetes*-dominated microbiota [11,13,14]. Disruption of T2R signaling, including downregulation of TAS2R108/TAS2R138 or impaired Gα-gustducin coupling, leads to reduced α-defensin secretion, dysbiosis, and compromised barrier integrity [15,16,17,18,19,20,21,22,23]. Chronic exposure to bitter alkaloids, such as berberine, can exacerbate gastrointestinal dysfunction by overactivating T2Rs [18].

Given DM’s intense bitterness, it remains unclear whether its components act as agonists or antagonists of T2Rs, potentially disrupting Paneth cell function. We hypothesize that DM interferes with the T2R108/T2R138–Gα-gustducin axis, suppressing α-defensin secretion, altering gut microbiota composition, degrading tight junctions, increasing intestinal permeability, and triggering low-grade inflammation, ultimately causing gastrointestinal adverse effects. This study aims to test this hypothesis through in vivo and in vitro experiments, providing mechanistic insight and a basis for the safe clinical use of DM.

## 2. Materials and Methods

### 2.1. Materials

A total of five kilograms of dried DM stems was pulverized into coarse powder. The powder was extracted three times with distilled water under reflux conditions, each extraction lasting 3 h. The combined aqueous extracts were filtered and concentrated under reduced pressure using a rotary evaporator (RE-2010, Shanghai Yaote Instrument Equipment Co., Ltd., Shanghai, China). The concentrated extract was subsequently freeze-dried at 60 °C using a freeze dryer (FD-1A-50, Beijing BoMedicom Experimental Instrument Co., Ltd.,(Beijing, China) to obtain the DM water extract. For animal experiments, the DM extract powder was suspended in 0.5% carboxymethylcellulose sodium (CMC-Na) solution and administered orally.

### 2.2. Animals and Ethics Statement

Six to eight-week-old male KM mice were purchased from Sibefu (Beijing) Biotechnology Co., Ltd. (Beijing, China), and five- to six-week-old male SD rats were obtained from Changsha Tianqin Biotechnology Co., Ltd. (Changsha, China). All animals were acclimated for one week prior to experiments with free access to water and food. All procedures were approved by the Ethics Committee of Hainan Medical University and complied with the Guideline on the Humane Treatment of Laboratory Animals issued by the Ministry of Science and Technology of China.

### 2.3. Grouping and DM Treatment

This study employed two parallel animal models to comprehensively assess the gastrointestinal effects of DM. The clinical oral dose of DM for a typical adult (70 kg) is 3–6 g once daily. For this study, the upper limit of this range (6 g/day, equivalent to ≈85.7 mg/kg) was used as the human equivalent dose (HED) for interspecies extrapolation. Animal doses were calculated based on body surface area normalization, using species-specific conversion factors derived from Pharmacological Experimental Methodology [24,25,26]. According to the standard conversion table, the equivalent dose for rats is 6.3 times the human dose on a mg/kg basis, and for mice it is 9.1 times.

**Figure 1:**
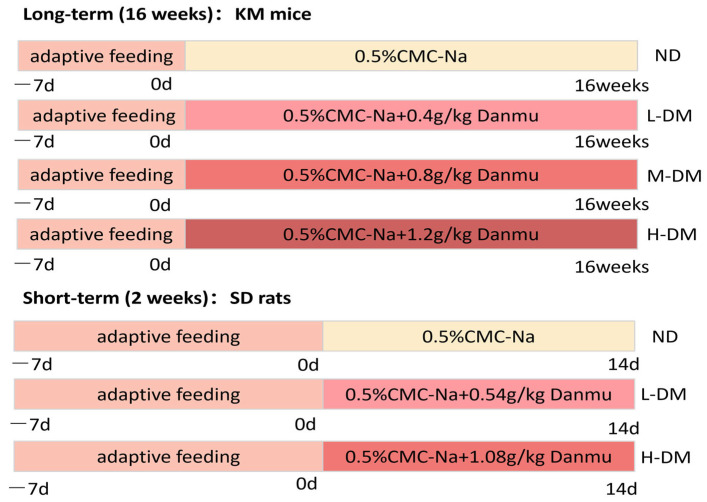
Schematic experimental design.

To explore the potential toxicity boundary and acute responses, a short-cycle (2 weeks) exposure model was established in SD rats [27]. Twenty-four SD rats were randomly divided into 3 groups (*n* = 8 per group): normal control (ND), low-dose DM (L-DM, 0.54 g/kg), and high-dose DM (H-DM, 1.08 g/kg). These doses correspond to approximately 1.0× and 2.0× the HED, respectively. Conversely, to simulate more realistic, long-term exposure and observe chronic adaptations or injuries, a long-cycle (16 weeks) exposure model was implemented in KM mice [28,29,30]. Forty KM mice were initially selected, with 8 allocated for in vitro intestinal experiments. The remaining 32 mice were randomly divided into 4 groups (n = 8 per group): normal control (ND), low-dose DM (L-DM, 0.4 g/kg), medium-dose DM (M-DM, 0.8 g/kg), and high-dose DM (H-DM, 1.2 g/kg). These doses correspond to approximately 0.5×, 1.0×, and 1.5× of the HED, respectively. All animals received daily intragastric gavage of the corresponding DM suspension or vehicle. The overall experimental design is summarized in Figure 1.

### 2.4. Enzyme-Linked Immunosorbent Assay (ELISA)

Serum levels of TNF-α, IL-1β, IL-6, MDA, SOD, IFN-γ, GSH-Px, and α-defensin were determined using commercial assay kits (Fankewei, Shanghai, China) according to the manufacturer’s protocols.

### 2.5. Intestinal Flora Diversity

After euthanasia, cecal contents were collected into cryovials and subjected to 16S rRNA sequencing for gut microbiota analysis. This sequencing service was commissioned to Wuhan Metware Technology Co., Ltd. (Wuhan, China). In short, cecal fecal samples were collected and immediately stored at −80 °C. Genomic DNA was extracted using the CTAB method. The V3–V4 region of the bacterial 16S rRNA gene was amplified with primers 341F (5′-CCTAYGGGRBGCASCAG-3′) and 806R (5′-GGACTACNNGGGTATCTAAT-3′). PCR products were pooled, purified, and used for library construction with the TruSeq^®^ DNA PCR-Free Kit. After quantification by Qubit 2.0 and qPCR, libraries were sequenced on the NovaSeq 6000 platform (Metware Technology Co., Ltd., Wuhan, China). Raw reads were demultiplexed and trimmed to remove barcode and primer sequences, and paired-end reads were merged using FLASH (v1.2.7). Quality filtering and chimera removal were performed in QIIME (v1.9.1) to obtain high-quality effective tags. OTU clustering was conducted using Uparse (v7.0.1001) at 97% similarity. Taxonomic assignment was performed with the Silva SSU rRNA database using the Mothur classifier. Alpha and beta diversity analyses were completed in QIIME, and community composition at different taxonomic levels was analyzed and visualized in R (v2.15.3).

### 2.6. Gastrointestinal Motility Assessment: Small Intestinal Propulsion and Gastric Retention Rate

On the final day of modeling and drug administration, rats were fasted for 12 h with free access to water. Subsequently, each rat received a 10 mL·kg^−1^ dose of semi-solid paste via oral gavage. The semi-solid paste was prepared as follows: 10 g of carboxymethyl cellulose sodium was dissolved in 250 mL of distilled water, followed by the addition of 16 g of milk powder, 8 g of sugar, 8 g of starch, and 3 g of activated carbon. The mixture was stirred uniformly to form 300 mL (approximately 300 g) of semi-solid paste, stored at 4 °C, and allowed to reach room temperature for 2 h before use. Thirty minutes after gavage, rats were anesthetized for sample collection. Blood was drawn from the abdominal aorta, and the abdominal cavity was opened. The pylorus and cardia were ligated with surgical sutures, and the stomach was excised. After removing residual blood with filter paper, the total stomach weight was recorded. The stomach was then opened along the greater curvature, and the contents were rinsed with physiological saline. The empty stomach was blotted dry with filter paper and weighed again to obtain the net weight. Gastric retention rate was calculated as (Total stomach weight − Net stomach weight)/Mass of administered semi-solid paste × 100%*; Gastric emptying rate was derived as [1 − (Total stomach weight − Net stomach weight)/Mass of administered semi-solid paste] × 100%. Following stomach collection, the intestine was isolated. The segment from the pylorus to the ileocecal junction was carefully separated and measured to determine the total small intestinal length. The distance from the pylorus to the farthest point of charcoal advancement was measured to assess propulsion. Small intestinal propulsion rate was calculated as (Distance from pylorus to charcoal forefront/Total small intestinal length) × 100% [31].

### 2.7. RNA Isolation and Real-Time PCR

Total RNA was extracted from mouse ileum tissues using the Eastep^®^ Super Total RNA Extraction Kit, and its concentration was measured with a BIO-RAD spectrophotometer. The RNA was then reverse transcribed into cDNA using the Hifair^®^ III 1st Strand cDNA Synthesis SuperMix for qPCR (gDNA digester plus) (Yeasen Biotechnology (Shanghai) Co., Ltd., Shanghai, China). The resulting cDNA products were used as templates for quantitative PCR (qPCR), which was performed on a BIO-RAD qPCR detection system with Hifair^®^ qPCR SYBR Green Master Mix (Low Rox Plus) (Yeasen Biotechnology (Shanghai) Co., Ltd., Shanghai, China). Relative gene expression was analyzed using the 2^(−ΔΔCT)^ method, with β-actin serving as the internal reference gene. Primer sequences are listed in Table 1.

### 2.8. Western Blot Assay

Total protein was extracted from ileal tissues using RIPA lysis buffer (strong) supplemented with 1% phenylmethylsulfonyl fluoride (PMSF). The protein concentration was measured using a protein assay kit according to the manufacturer’s protocol. The samples were then diluted to equal concentrations and denatured with 5× SDS loading buffer at 90 °C for 10 min. The proteins were separated by electrophoresis on 10% SDS-polyacrylamide gels and transferred onto PVDF membranes. Subsequently, the membranes were blocked by incubation in Rapid Blocking Buffer at room temperature for 1 h, followed by incubation with primary antibodies overnight at 4 °C. After washing three times with Tris-buffered saline containing Tween-20 (TBST), the membranes were incubated with secondary antibodies at room temperature for 2 h. All information regarding the primary and secondary antibodies is listed in Table 2. The protein bands were visualized using an ECL Plus kit, and the signals were captured using a Bio-Rad ChemiDoc XRS+ molecular imager system. The intensity of each band was analyzed using Image Lab software (version 5.2.1, Bio-Rad, Hercules, CA, USA). GAPDH protein was used as a reference for normalizing the results of the target proteins. The final results for each target protein are expressed as its abundance relative to that of GAPDH.

### 2.9. Hematoxylin and Eosin Staining

The terminal ileum and proximal colon were collected and fixed in 4% paraformaldehyde solution for 24 h. After paraffin embedding performed by Wuhan Servicebio Technology Co., Ltd. (Wuhan, China), the samples were sectioned into 3 μm thick slices using a microtome. Intestinal damage was evaluated following hematoxylin and eosin staining.

### 2.10. Cells and Treatment

The enteroendocrine STC-1 cell line was purchased from iCell Bioscience (Shanghai) Inc. (Shanghai, China). Cells were maintained in culture medium supplemented with 20% fetal bovine serum and 1% penicillin-streptomycin solution, and incubated at 37 °C in a humidified atmosphere containing 5% CO_2_.

### 2.11. Cell Counting Kit-8 (CCK-8)

After counting, STC-1 cells were seeded into 96-well plates at a density of 7.5 × 10^4^ cells per well. Each well received 100 μL of culture medium, and the plates were incubated at 37 °C in a 5% CO_2_ atmosphere for 24 h. The original culture medium was then discarded, and treatments were administered to the respective groups: *Nauclea officinalis* extract (5 mg/mL) and streptomycin (STR, 1 mg/mL). Following 48 h of stimulation, the medium was removed, and the CCK-8 working solution was diluted 10-fold. A volume of 100 μL of the diluted CCK-8 solution was added to each well, and the plates were incubated for 2 h at 37 °C in a 5% CO_2_ incubator. Finally, the absorbance of each well was measured at a single wavelength of 450 nm.

### 2.12. Primary Intestinal Cell Culture

After one week of acclimatization feeding, Kunming mice were euthanized. The ileum (5 cm proximal to the ileocecal valve) was collected, and intestinal segments were stored in ice-cold KRB/HEPES buffer bubbled with O_2_/CO_2_ (95%/5%). For primary cell culture, the intestinal segments were longitudinally opened and cleared of debris using buffered KRB/HEPES. Each segment was then trimmed into 1 cm pieces. The circular tissue specimens were transferred to 12-well plates containing 1 mL of ice-cold KRB/HEPES buffer (pH 7.4) and maintained at room temperature for 30 min, followed by incubation in a humidified atmosphere at 37 °C with 5% (*v*/*v*) CO_2_. After 1 h of pre-incubation, the buffer was replaced with pre-warmed drug-containing KRB/HEPES buffer, and incubation was continued for an additional hour [32,33].

### 2.13. Lactate Dehydrogenase (LDH) Release Assay

LDH release was measured using an LDH assay kit (Shanghai Puyinte Bio Technology Co., Ltd., Shanghai, China)according to the manufacturer’s instructions. Briefly, after drug treatment, the isolated intestinal tissues were homogenized and centrifuged at 8500 rpm and 4 °C for 10 min. The tissue was then collected for analysis. The subsequent detection procedures were performed following the manufacturer’s protocol (Puyinte, Shanghai, China) [34].

### 2.14. Statistical Analysis

Statistical analysis was performed using GraphPad Prism software (version 9.5). Data are presented as mean ± standard error of the mean. All quantitative data were analyzed using one-way analysis of variance (ANOVA) for intergroup comparisons when homogeneity of variance was satisfied. When the assumption of homogeneity of variance was violated, the rank transformation test was employed. A *p*-value of less than 0.05 (*p* < 0.05) was considered statistically significant.

## 3. Results

### 3.1. Effects of Short-Term DM Intervention on Intestinal Function in Rats

#### 3.1.1. Effects of Short-Term DM Intervention on Gastrointestinal Motility in Rats

**Figure 2:**
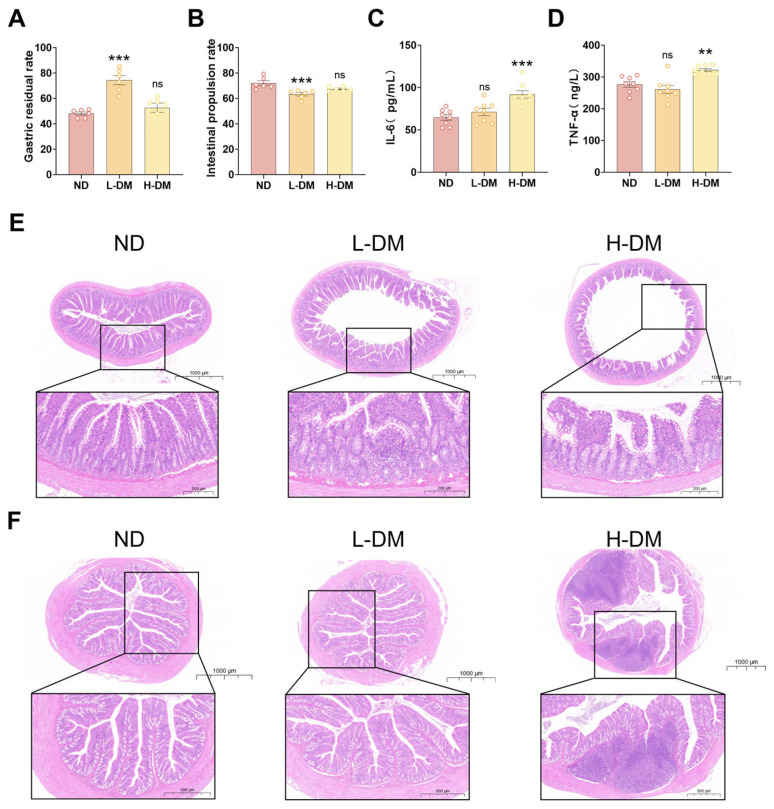
Effects of short-term DM intervention on intestinal morphology and function in rats: (**A**) gastric retention rate; (**B**) small intestinal propulsion rate; (**C**) serum IL-6 levels; (**D**) serum TNF-α levels; (**E**) representative H&E-stained images of ileal morphology (scale bar = 200 μm); (**F**) representative H&E-stained images of colonic morphology (scale bar = 500 μm). Data are means ± SEM; ns, not significant; ** *p* < 0.01, *** *p* < 0.001; one-way ANOVA. *n* = 6~8 animals/group.

To assess the effects of short-term DM intervention (14 days) on gastrointestinal motility, we measured the small intestinal propulsion rate (Figure 2A) and gastric retention rate (Figure 2B). Gastrointestinal motility disorders are typically characterized by reduced intestinal propulsion and delayed gastric emptying, often leading to symptoms such as reflux, diarrhea, and gastric retention [35]. Compared with the normal diet (ND) group, the low-dose DM (L-DM) group exhibited a significantly decreased small intestinal propulsion rate (*p* < 0.05), whereas the high-dose DM (H-DM) group showed a non-significant reduction (*p* > 0.05). Similarly, gastric retention was significantly elevated in the L-DM group (*p* < 0.05), while the H-DM group displayed a non-significant upward trend (*p* > 0.05).

#### 3.1.2. Effects of Short-Term DM Intervention on Inflammatory Levels in Rats

To assess intestinal inflammation following short-term DM intervention (14 days), serum levels of IL-6 (Figure 2C) and TNF-α (Figure 2D) were measured, as these are well-established biomarkers of drug-induced inflammatory responses [36]. Compared with the ND group, the L-DM group showed no significant changes in IL-6 or TNF-α levels. In contrast, the H-DM group exhibited significantly elevated levels of both cytokines (*p* < 0.05), indicating that high concentrations of DM may provoke inflammatory responses.

#### 3.1.3. Effects of Short-Term DM Intervention on the Morphology of the Ileum and Colon in Rats

As shown in Figure 2E, H&E staining of the ileum revealed intact villus architecture in the ND group, with well-organized columnar epithelial cells, preserved cellular junctions, and abundant submucosal connective tissue with orderly vasculature. The L-DM group exhibited focal epithelial detachment and reduced crypt numbers, including partial crypt atrophy and fusion. In the H-DM group, scattered inflammatory cell infiltration was observed in the lamina propria, accompanied by distorted villus morphology, cellular degeneration, and necrosis.

H&E staining of the colon (Figure 2F) showed that the ND group maintained orderly mucosal epithelium, regular crypt architecture, normal goblet cell morphology, and absence of inflammatory infiltration. The L-DM group retained basic mucosal integrity with occasional epithelial exfoliation and mild crypt disorganization without significant edema. In contrast, the H-DM group displayed dense inflammatory cell infiltration—including neutrophils, lymphocytes, and macrophages—throughout the lamina propria, with extensive formation of inflammatory foci.

### 3.2. Effects of Long-Term DM Intervention on the Intestine in Mice

#### 3.2.1. Effects of Long-Term DM Intervention on Intestinal Inflammatory Levels in Mice

**Figure 3:**
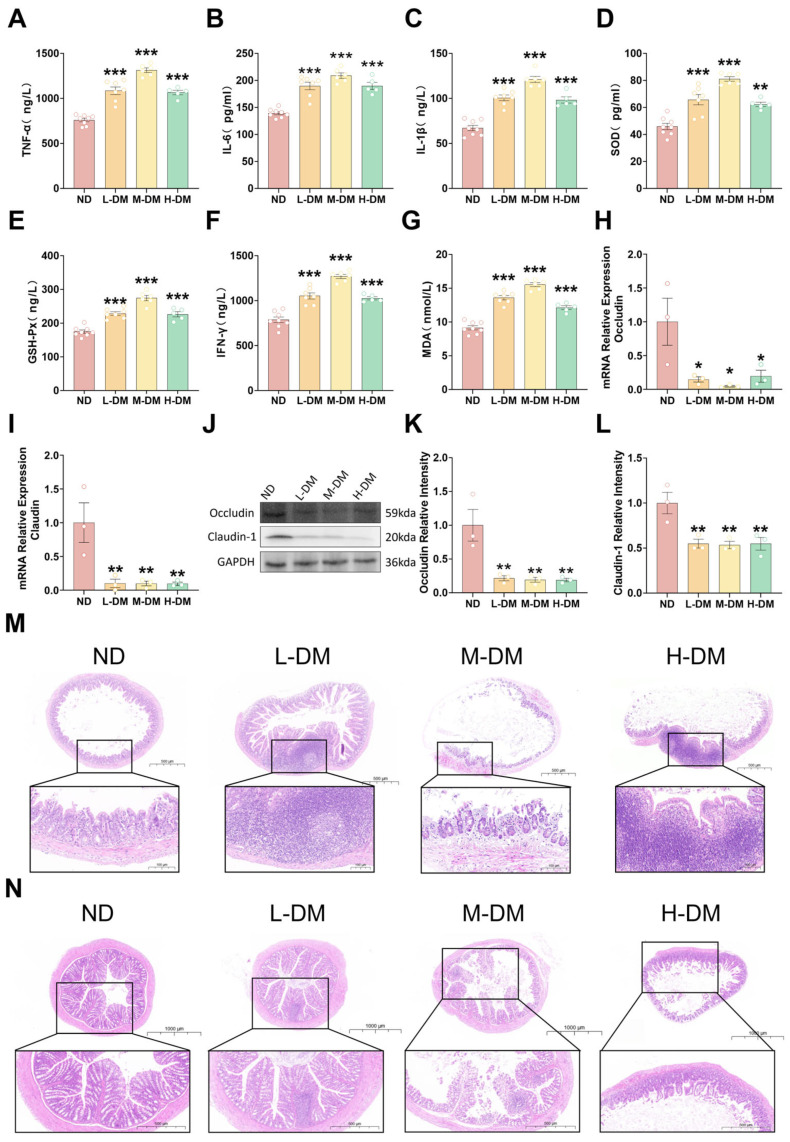
Effects of long-term DM intervention on the intestinal tract in mice: (**A**) serum TNF-α levels; (**B**) serum IL-6 levels; (**C**) serum IL-1β levels; (**D**) serum SOD levels; (**E**) serum GSH-Px levels; (**F**) serum IFN-γ levels; (**G**) serum MDA levels; (**H**) relative mRNA expression of occludin in ileal tissues; (**I**) relative mRNA expression of claudin-1 in ileal tissues; (**J**) Western blot analysis of Occludin and claudin-1 protein levels in ileal tissue extracts; (**K**) relative protein abundance of Occludin in ileal tissues, normalized to GAPDH; (**L**) relative protein abundance of claudin-1 in ileal tissues, normalized to GAPDH; (**M**) representative H&E-stained images of ileal morphology (scale bar = 100 μm); (**N**) representative H&E-stained images of colonic morphology (scale bar = 500 μm). Data are means ± SEM; * *p* < 0.05, ** *p* < 0.01, *** *p* < 0.001; one-way ANOVA. *n* = 5~8 animals/group for ELISA assays. *n *= 3 animals/group for qRT-PCR and Westen blot analysis.

To assess intestinal inflammation following a 16-week DM intervention, serum levels of IL-6, TNF-α, IL-1β, IFN-γ, GSH-Px, SOD, and MDA were measured. Compared with the normal control group, DM-treated animals exhibited significantly elevated levels of all these markers (*p* < 0.05; Figure 3A–G). These results indicate that long-term DM administration triggers systemic inflammatory responses, as evidenced by increased IL-6, TNF-α, IL-1β, and IFN-γ, and induces oxidative stress, reflected by elevated MDA levels accompanied by compensatory changes in SOD and GSH-Px.

#### 3.2.2. Effects of Long-Term DM Intervention on Intestinal Wall Integrity in Mice

To assess intestinal barrier integrity following long-term DM intervention (16 weeks), we determined the relative expression levels of occludin and claudin using Western blot and RT-PCR analyses. The RT-PCR results revealed that compared with the ND group, all DM-treated groups exhibited significantly reduced expression of both occludin (Figure 3H) and claudin-1 (Figure 3I) (*p* < 0.05). Consistent with these findings, Western blot analysis demonstrated that the protein expression levels of occludin (Figure 3J,K) and claudin-1 (Figure 3J,L) were also significantly decreased in all treatment groups compared to the ND group (*p* < 0.05).

#### 3.2.3. Effects of Long-Term DM Intervention on the Morphology of the Ileum and Colon in Mice

H&E staining revealed dose-dependent intestinal injury (Figure 3M,N). In the ileum, the ND group showed mild villus shortening with intact crypts; the L-DM group exhibited localized inflammation, partial villus fusion, and crypt disruption; the M-DM group displayed extensive villus loss, severely damaged crypts, mucosal edema, and obscured vasculature; the H-DM group showed diffuse inflammatory infiltration, complete villus destruction, and unrecognizable crypts.

In the colon, the ND group maintained well-organized mucosa with intact crypts and no inflammation; the L-DM group had focal epithelial exfoliation and mild crypt disorganization; the M-DM group exhibited multiple erosions, irregular epithelial morphology, and lamina propria inflammation; the H-DM group showed extensive epithelial necrosis, crypt abscesses, and widespread inflammatory infiltration. These results indicate that DM induces dose-dependent intestinal injury, progressing from mild architectural changes to severe epithelial destruction.

### 3.3. Effects of Long-Term DM Intervention on Intestinal Flora

#### 3.3.1. Effects of DM on Alpha and Beta Diversity

**Figure 4:**
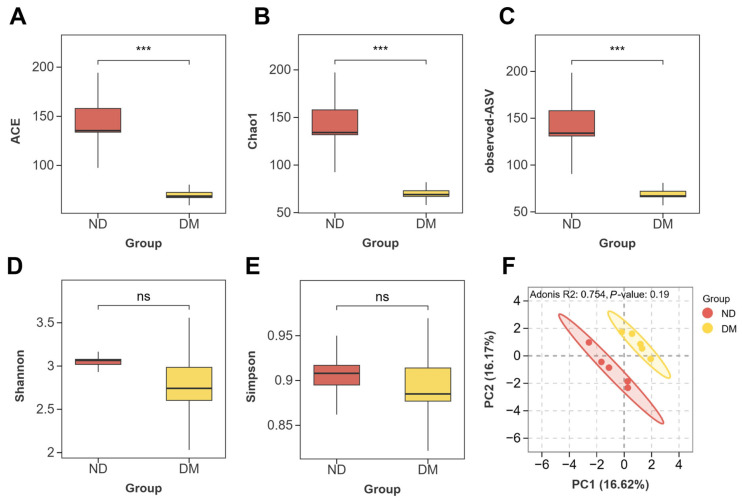
Effects of long-term DM intervention on α- and β-diversity of gut microbiota in mice. parameters of α-diversity: (**A**) ACE index; (**B**) Chao1 index; (**C**) observed ASVs; (**D**) Shannon index; (**E**) Simpson index; (**F**) principal coordinate analysis (PCoA) plot of gut microbiota. Data are means ± SEM; ns, not significant; *** *p* < 0.001; one-way ANOVA. *n* = 5 animals/group.

To investigate the effects of DM on the gut microbiota, we analyzed both α- and β-diversity. The M-DM group was selected for 16S rRNA sequencing. As shown in Figure 4, compared with the ND group, the DM-treated (DM) group exhibited significantly decreased Chao1 (Figure 4A), ACE (Figure 4B), and observed ASVs (Figure 4C) indices (*p* < 0.05). Although the Simpson (Figure 4D) and Shannon (Figure 4E) indices also showed a decreasing trend, the differences were not statistically significant (*p* > 0.05). In the principal coordinate analysis (PCoA) plot (Figure 4F), clear separation was observed between the ND and DM groups, indicating that DM treatment reduced microbial species richness and diversity, and altered the overall structure of the gut microbiota in mice.

#### 3.3.2. Effects of DM on the Composition of Intestinal Microorganisms

**Figure 5:**
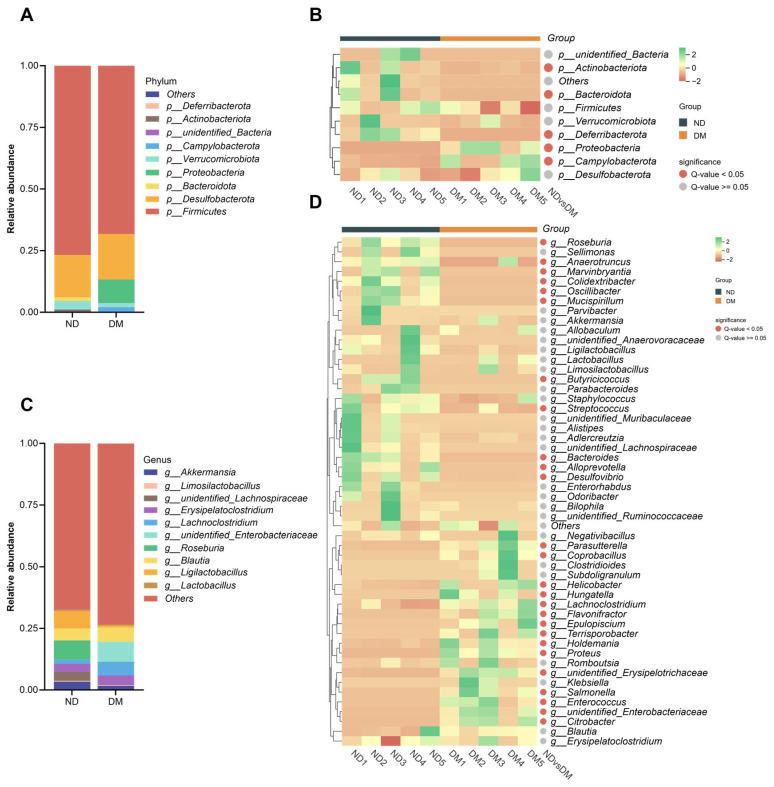
Effects of long-term DM intervention on gut microbial composition: (**A**) histogram of species composition at the phylum level; (**B**) heatmap of species composition at the phylum level; (**C**) histogram of species composition at the genus level; (**D**) heatmap of species composition at the genus level. *n* = 5 animals/group.

Following DM administration, significant alterations in the gut microbial composition at the phylum level were observed (Figure 5A,B). Compared with the ND group, the DM group exhibited significantly increased abundances of Proteobacteria and Campylobacterota (*p* < 0.05), while the abundances of Bacteroidota, Actinobacteriota, and Deferribacterota were significantly decreased (*p* < 0.05).

At the genus level (Figure 5C,D), the DM group showed significantly reduced abundances of Bacteroides, Alloprevotella, Anaerotruncus, Butyricicoccus, Colidexeribacter, Desulfovibrio, Marvinbryantia, Mucispirillum, Oscillibacter, Roseburia, and Streptococcus (*p* < 0.05). Conversely, significantly increased abundances were observed for Citrobacter, Coprobacillus, Enterococcus, Epulopiscium, Flavonifractor, Helicobacter, Holdemania, Hungatella, Lachnoclostridium, Parasutterella, Proteus, Salmonella, Terrisporobacter, unidentified Enterobacteriaceae, and unidentified Erysipelotrichaceae (*p* < 0.05).

#### 3.3.3. Correlation Analysis Between Gut Microbiota and Inflammatory/Oxidative Stress Biomarkers

**Figure 6:**
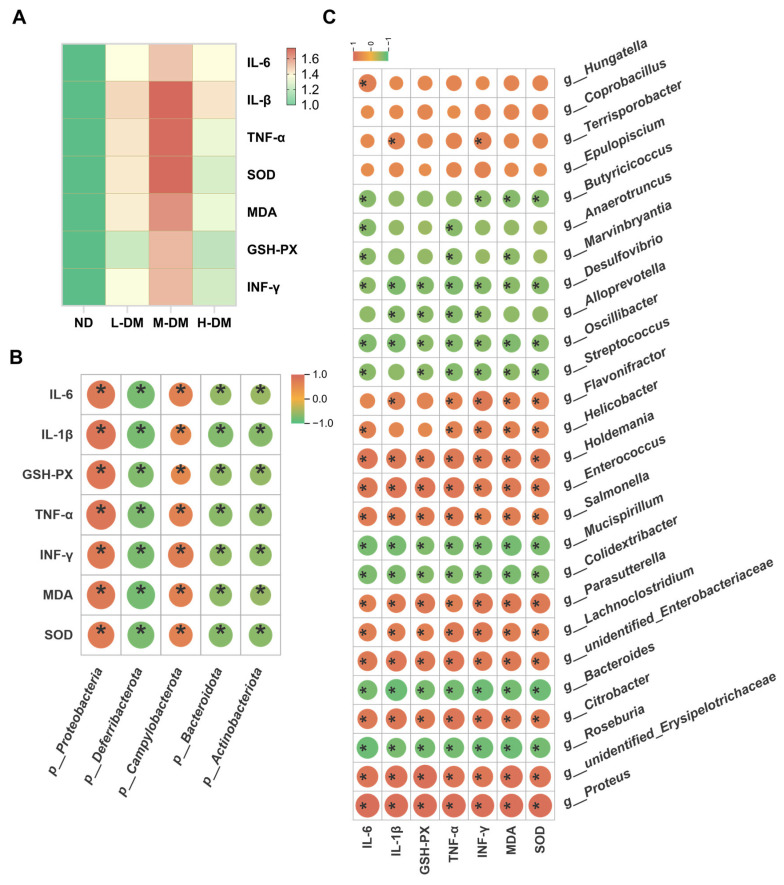
Correlation analysis between gut microbiota alterations and inflammatory/oxidative stress biomarkers: (**A**) Heatmap of inflammatory/oxidative stress biomarker profiles. Normalization was performed using the ND group as the reference (set to 1). Values > 1 indicate upregulation, with increasingly dusty red hues representing stronger upregulation. (**B**) Correlation analysis between inflammatory/oxidative stress biomarkers and gut microbiota at the phylum level. In the figure legend, colors represent the magnitude of Spearman rank correlation coefficients: ρ = 1 (dusty red) indicates a perfect positive correlation, ρ = −1 (mint green) represents a perfect negative correlation, and ρ = 0 (sandy yellow) denotes no correlation. (**C**) Correlation analysis between inflammatory/oxidative stress biomarkers and gut microbiota at the genus level. In the figure legend, colors represent the magnitude of Spearman rank correlation coefficients: ρ = 1 (dusty red) indicates a perfect positive correlation, ρ = −1 (mint green) represents a perfect negative correlation, and ρ = 0 (sandy yellow) denotes no correlation. Statistical annotations: ns, not significant; * *p* < 0.05. *n* = 5 animals/group for correlation analysis.

To investigate the correlation between gut microbiota alterations and inflammatory/oxidative stress biomarkers, we performed correlation analyses of microbial changes at both the phylum and genus levels with these host factors in a mouse model. As illustrated in Figure 6, all DM-treated groups exhibited significantly elevated levels of inflammatory/oxidative stress biomarkers compared to the normal control group (Figure 6A). Analysis at the phylum level (Figure 6B) revealed positive correlations between these biomarkers and the abundances of Proteobacteria and Campylobacterota, while negative correlations were observed with Bacteroidota, Actinobacteriota, and Deferribacterota. At the genus level (Figure 6C), the biomarkers showed significant negative correlations with several commensal and beneficial genera, including Bacteroides, Alloprevotella, Butyricicoccus, Roseburia, and Oscillibacter, but positive correlations with multiple opportunistic or pathogenic taxa such as Helicobacter, Salmonella, Citrobacter, Enterococcus, and Lachnoclostridium. These results indicate that the pro-inflammatory effects induced by DM are closely associated with gut microbial dysbiosis.

### 3.4. Effects of DM on Cell/Tissue Viability and T2R/α-Defensin Expression

#### 3.4.1. Effects of DM on Viability in STC-1 Cells and Ileal Ex Vivo Tissue

**Figure 7:**
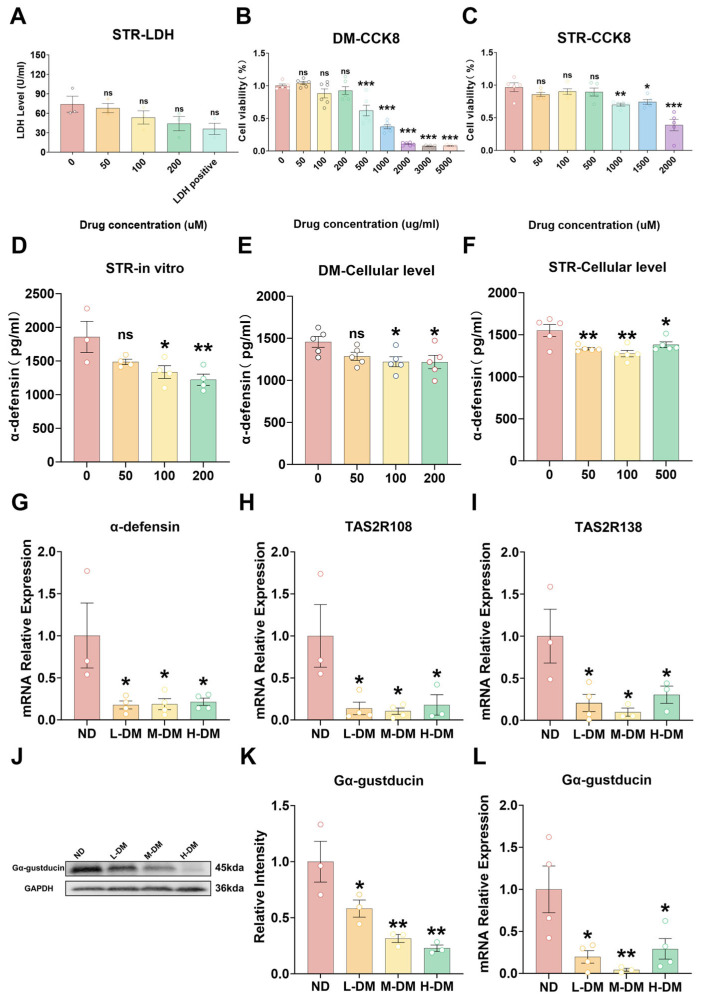
Effects of DM on Cell/Tissue Viability and T2R/α-Defensin Expression. (**A**) Impact of strictosamide on the viability of ex vivo ileal tissues. (**B**) Effect of DM extract on STC-1 cell viability. (**C**) Influence of strictosamide on STC-1 cell viability. (**D**) α-defensin secretion level in ex vivo ileal tissues. (**E**) α-defensin secretion level in STC-1 cells after treatment with DM extract. (**F**) α-defensin secretion level in STC-1 cells after treatment with strictosamide. (**G**) Relative mRNA expression of α-defensin in ileal tissues. (**H**) Relative mRNA expression of TAS2R108 in ileal tissues. (**I**) Relative mRNA expression of TAS2R138 in ileal tissues. (**J**) Western blot analysis of Gα-gustducin protein levels in ileal tissue extracts. (**K**) Relative protein abundance of Gα-gustducin in ileal tissues normalized to GAPDH. (**L**) Relative mRNA expression of Gα-gustducin in ileal tissues. Data are means ± SEM; ns, not significant; * *p* < 0.05, ** *p* < 0.01; *** *p* < 0.001; one-way ANOVA. *n* = 3~4 animals/group for qRT-PCR and Westen blot analysis. ELISA, LDH and CCK-8 assays were performed with *n* = 3~6 wells/concentration.

To assess the effects of DM on STC-1 cell viability and intestinal tissue integrity, we employed the CCK-8 assay for STC-1 proliferation and metabolic activity, and the LDH release assay for ex vivo ileal tissue viability. While strictosamide induced a concentration-dependent decrease in LDH release, the effect was not statistically significant (Figure 7A, *p* > 0.05). In contrast, DM extract caused a dose-dependent reduction in STC-1 viability over 48 h. Specifically, exposure to 1000 μg/mL DM extract or 1500 μM strictosamide reduced cell viability below 80% (Figure 7B,C, *p* < 0.05), commonly accompanied by compromised membrane integrity, mitochondrial dysfunction, oxidative stress, and activation of apoptotic/necrotic pathways. Subsequent in vitro experiments were conducted using sub-cytotoxic concentrations of DM extract (0–200 μg/mL) and strictosamide (0–500 μM) to avoid confounding effects from overt cytotoxicity.

#### 3.4.2. Effects of DM on the Expression of Antimicrobial Peptides

To assess the impact of DM on antimicrobial peptide secretion, α-defensin expression and release were evaluated in vivo, in vitro, and ex vivo. Ex vivo, ELISA demonstrated that strictosamide at 100 and 200 μM markedly suppressed α-defensin secretion relative to controls (Figure 7D). In STC-1 cells, both DM extract and strictosamide inhibited α-defensin release: 100 and 200 μg/mL DM extract significantly reduced secretion (Figure 7E), and all tested concentrations of strictosamide produced significant decreases compared with controls (Figure 7F). RT-qPCR showed that DM treatment significantly reduced α-defensin mRNA expression compared with the ND group (Figure 7G).

#### 3.4.3. Effects of DM on the Expression of Bitter Taste Receptors

To investigate the effects of DM on bitter taste receptor expression, we analyzed the relative expression levels of intestinal TAS2R108 (Figure 7H), TAS2R138 (Figure 7I), and Gα-gustducin (Figure 7L) by RT-qPCR, and detected the protein expression of Gα-gustducin by Western blot (Figure 7J,K). The RT-qPCR results demonstrated that compared with the normal control group, DM-treated groups showed significantly lower relative expression levels of TAS2R108, TAS2R138, and Gα-gustducin (*p* < 0.05). Consistent with these findings, Western blot analysis revealed that the protein expression of Gα-gustducin was also significantly downregulated in all treatment groups compared to the normal control group (*p* < 0.05; Figure 7J,K). Our results demonstrate that DM treatment significantly suppressed the expression of intestinal TAS2R108, TAS2R138, and Gα-gustducin in mice (Figure 7).

## 4. Discussion

This study provides a systematic and mechanistic framework for understanding the gastrointestinal toxicity associated with *Nauclea officinalis* (DM), addressing a long-standing gap in the toxicological evaluation of traditional medicinal plants. Although DM has demonstrated therapeutic potential, accumulating clinical observations have reported gastrointestinal adverse reactions [3,37], yet the initiating molecular events and downstream pathogenic pathways remain largely undefined. Our findings reveal that DM induces gastrointestinal injury through distinct mechanisms under short-term and long-term exposure, ultimately converging on impairment of intestinal motility, disruption of mucosal barrier integrity, innate immune suppression, and dysregulation of bitter taste receptor-mediated signaling.

Short-term toxicity evaluations demonstrated a concentration-dependent effect of DM. In vitro assays indicated that the threshold concentration for reducing cell viability below 80% was approximately 1000 μg/mL for DM extract and 1500 μM for strictosamide; however, the ex vivo ileum model showed limited sensitivity, likely due to complex tissue-level compensatory factors. In vivo experiments revealed that low-dose DM impaired gastrointestinal motility, a pattern consistent with the motility disorders associated with irritable bowel syndrome (IBS), inflammatory bowel disease (IBD), and peptic ulcer disease [38,39,40]. The low-dose effect observed after short-term exposure may also involve hormesis or compensatory mechanisms, in which DM acts as a mild stressor, interfering with neural and muscular regulation. In contrast, high-dose DM induced systemic inflammation, characterized by elevated levels of IL-6 and TNF-α, as well as early mucosal damage in the ileum and colon—a finding consistent with previously reported inflammatory injury caused by high-dose botanical preparations [41,42]. These results highlight a potential dose-threshold effect in DM toxicity. At low doses, DM did not elicit significant inflammation, which may be attributed to the preservation of barrier function and antioxidant homeostasis (e.g., via the Nrf2 pathway) [43]. However, once the dose exceeds the threshold, cumulative DM overwhelms the defense mechanisms, activates inflammatory pathways (such as NF-κB), and may mask symptoms of motility dysfunction [44]. Such non-monotonic dose–response patterns, including hormesis and compensation–decompensation transitions, have been reported in natural product toxicology and are consistent with a two-phase toxicity framework characterized by early functional adaptation followed by pathological injury [45,46,47,48].

To explore whether these acute perturbations progress toward chronic gastrointestinal dysfunction, we extended our investigation to long-term exposure models. Chronic DM administration resulted in a marked deterioration of intestinal barrier integrity, characterized by significant downregulation of the tight junction proteins occludin and claudin-1 [49,50,51,52]. This structural and functional impairment of the tight junction complex mechanistically explains the dysregulated osmotic balance observed in our model [53,54].

A major discovery of this study is the profound effect of long-term DM intervention on the gut microbial ecosystem. DM induced an expansion of *Proteobacteria* and other opportunistic pathogens [55,56,57], accompanied by depletion of beneficial taxa such as *Bacteroidetes* [58], *Actinobacteria* [59,60], and butyrate-producing genera including *Roseburia* and *Faecalibacterium*. Given that butyrate regulates epithelial energy metabolism, junctional protein expression, and mucus secretion [61,62], its decline likely contributes to DM-induced barrier dysfunction.

Consistent with these observations, long-term DM exposure induced systemic inflammation (IL-6, TNF-α, IL-1β, IFN-γ) and oxidative stress (altered SOD, GSH-Px, and elevated MDA) [63]. This supports a progressive pathological model in which gut dysbiosis and barrier disruption trigger systemic inflammatory and oxidative responses that further damage the intestinal epithelium—processes known to involve lipid peroxidation and protein oxidation [64], as well as NF-κB–mediated MLCK activation leading to tight junction destabilization [65]. Correlation analyses reinforced this model, revealing positive associations between pathogenic taxa and inflammatory/oxidative markers, and negative associations with beneficial microbes. These findings align with the “dysbiosis → barrier impairment → systemic inflammation/oxidative stress → worsened barrier dysfunction” hypothesis [66].

Beyond microbiota-mediated pathways, this study highlights the crucial role of innate immunity, particularly α-defensins, in DM-induced gastrointestinal toxicity. Through in vivo, ex vivo, and in vitro models, we confirmed that DM suppresses α-defensin expression and secretion [11,67]. Since α-defensins inhibit opportunistic pathogens and maintain microbial homeostasis, their reduction provides a mechanistic explanation for the observed dysbiosis and tight junction impairment.

Importantly, we found that the suppression of the bitter taste receptor signaling axis (TAS2R108/TAS2R138–Gα-gustducin) may be a central underlying mechanism of long-term DM toxicity. Dysfunction of T2R signaling has been linked to reduced defensin secretion [68], microbial dysbiosis, and worsened colitis in DSS-induced models [15]. TAS2R138 deficiency has also been associated with impaired immune function and defensin regulation [69]. Notably, unlike other bitter phytochemicals such as constituents of Coptis chinensis that act via T2R activation, DM uniquely inhibits T2R signaling [19], highlighting a mechanistic divergence in bitter herb-induced enterotoxicity.

While DM has reported anti-infective activity [70], our findings emphasize its dual nature—therapeutic at appropriate doses but potentially harmful with long-term or excessive use. This underscores the importance of dose, duration, and safety evaluation in clinical applications of herbal medicines. Identification of the T2R pathway as a key regulatory node suggests potential therapeutic avenues; T2R agonists may restore defensin production and intestinal homeostasis and may serve as promising candidates for inflammatory bowel disease therapy.

Several limitations of this study warrant consideration, which concurrently delineate priorities for subsequent research. First, although ileal tissue was selected for mechanistic analysis based on its high T2R/α-defensin expression, and colonic pathology mirrored ileal changes, region-specific effects—particularly those mediated by the colonic microbiota—require further clarification. Second, the causal relationship between suppressed T2R signaling and the observed intestinal phenotypes remains to be functionally validated using, for instance, T2R-knockout models or specific receptor modulators. Third, as DM is a multi-component extract, the individual contribution of strictosamide must be dissected from potential synergistic or additive interactions with other constituents. Fourth, the lack of pharmacokinetic data regarding systemic and local intestinal exposure impedes a precise toxicokinetic interpretation of the dose-response relationships. Fifth, interspecies differences between the rat (acute) and mouse (chronic) models may affect the extrapolation of findings. Finally, despite stringent procedural controls, the extended 16-week gavage protocol entails inherent methodological constraints due to repeated mechanical stimulation. To address these limitations, future investigations should employ an integrated approach, combining T2R-deficient animal models with region-resolved transcriptomics (e.g., single-cell or spatial), targeted metabolomic profiling of DM components, and comprehensive pharmacokinetic–toxicodynamic analyses. Such strategies will be essential to verify and extend the mechanistic framework proposed herein.

## 5. Conclusions

**Figure 8:**
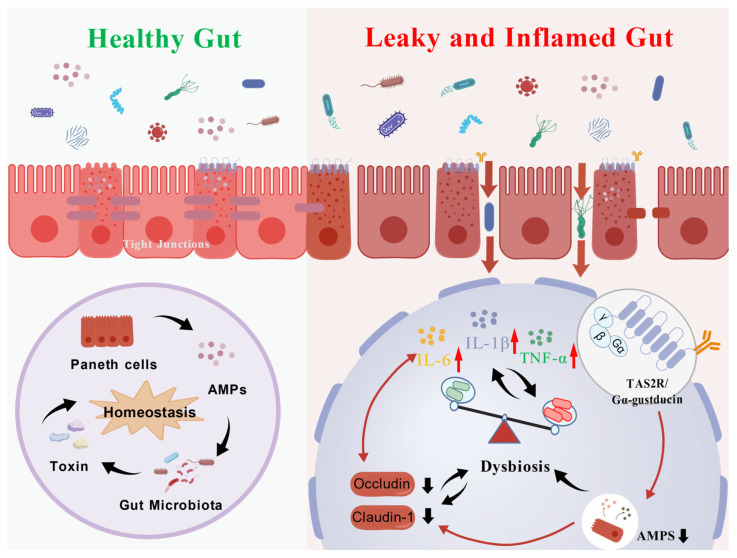
Proposed mechanism of *Nauclea officinalis*-induced gastrointestinal toxicity (Created in BioGDP. Li, X. (2026) https://BioGDP.com/GDP2026U27W7E).

In conclusion, this study systematically delineates the mechanistic basis of the gastrointestinal toxicity induced by both short-term and long-term exposure to *Nauclea officinalis*. Short-term administration predominantly resulted in gastrointestinal motility disturbances at low doses, while high-dose exposure provoked acute enteritis through the upregulation of pro-inflammatory cytokines such as IL-6 and TNF-α. In contrast, long-term toxicity was closely associated with the inhibition of intestinal bitter taste receptor signaling (specifically TAS2R108/TAS2R138 and Gα-gustducin). The data suggest that this downregulation contributes to marked suppression of enteric α-defensin secretion, gut microbiota dysbiosis, impairment of the mucosal barrier, and subsequent systemic inflammation (Figure 8). Collectively, our findings point to the bitter taste receptor signaling axis as a potential key mechanism underlying the chronic intestinal toxicity of this traditional medicinal herb, offering a new conceptual framework for understanding the enterotoxicity of bitter-cold herbal medicines and highlighting an important direction for future research on their safe clinical application.

## Figures and Tables

**Table 1:** List of primer sequences for quantitative real-time PCR.

Primer Name	Primer Name
Actb-1R	GACCCATTCCCACCATC
Actb-1F	TCTTTGCAGCTCCTTCGT
Defa-5-R	GCAGCCTCTTATTCTACAATAGCA
Defa-5-F	CTAATACTGAGGAGCAGCCAGG
Ocln-1F	CTGCCTGCACGATGTCT
Ocln-1R	GAGTGTTCAGCCCAGTCAA
Cldn11-2F	CAGGTGGTGGGTTTCGT
Cldn11-2R	CAGGTGGGGATGGTGTAG
Tas2r108-2F	AACAGGACCAGCTTTTGGAATC
Tas2r108-2R	GAGGAAACAGATCATCAGCCTCAT
Tas2r138-1F	CACAACTACCAAGCCATCC
Tas2r138-1R	TGTGAGAGAAGCGGACAA
Gnat3-1F	CCCAGCCACTAACATCAAA
Gnat3-1R	TTCACAGTTCTTGCATCCCT

**Table 2:** Details of antibodies used in Western blot and immunohistochemistry analyses.

Antibody	Product Code	Manufacturer	Dilution Ratio
GAPDH	AG8015	Beyotime, Shanghai, China	1:2000
Occludin	AF7644	Beyotime	1:500
Claudin-1	AF6504	Beyotime	1:500
Gα-gustducin	sc-518163	SANTA, Dallas, TX, USA	1:100

## Data Availability

The datasets generated and analyzed during the current study are available from the corresponding author upon reasonable request, subject to privacy protection and ethical requirements.

## Abbreviations

The following abbreviations are used in this manuscript:

DM	*Nauclea officinalis*
T2R	Bitter taste receptor
TNF-α	Tumor necrosis factor-alpha
IL-6	Interleukin-6
IL-1β	Interleukin-1 beta
IFN-γ	Interferon-gamma
SOD	Superoxide dismutase
MDA	Malondialdehyde
GSH-PX	Glutathione peroxidase
